# Oculomics meets exposomics: a roadmap for applying multi-modal ocular biomarkers in precision environmental health research

**DOI:** 10.1093/exposome/osaf013

**Published:** 2025-11-10

**Authors:** Haoran Cheng, Jeremy A Sarnat, Douglas I Walker, Anant Madabhushi, Amritpal Singh, Rohan Dhamdhere, Jodhbir S Mehta, Tien Yin Wong, John S Ji, Carmen J Marsit, Dean P Jones, Daniel S W Ting, Darren S J Ting, Donghai Liang

**Affiliations:** Singapore Eye Research Institute, Singapore National Eye Center, Singapore, Singapore; Duke-NUS Medical School, National University of Singapore, Singapore, Singapore; Gangarosa Department of Environmental Health, Rollins School of Public Health, Emory University, Atlanta, GA, United States; Gangarosa Department of Environmental Health, Rollins School of Public Health, Emory University, Atlanta, GA, United States; Department of Radiology and Imaging Sciences, Emory University School of Medicine, Emory University, Atlanta, GA, United States; Wallace H. Coulter Department of Biomedical Engineering, Emory University and GA Institute of Technology, Atlanta, GA, United States; Emory Empathetic AI for Health Institute, Emory University, Atlanta, GA, United States; Atlanta Veterans Administration Medical Center, Atlanta, GA, United States; Department of Radiology and Imaging Sciences, Emory University School of Medicine, Emory University, Atlanta, GA, United States; Wallace H. Coulter Department of Biomedical Engineering, Emory University and GA Institute of Technology, Atlanta, GA, United States; Department of Radiology and Imaging Sciences, Emory University School of Medicine, Emory University, Atlanta, GA, United States; Wallace H. Coulter Department of Biomedical Engineering, Emory University and GA Institute of Technology, Atlanta, GA, United States; Singapore Eye Research Institute, Singapore National Eye Center, Singapore, Singapore; Duke-NUS Medical School, National University of Singapore, Singapore, Singapore; Singapore Eye Research Institute, Singapore National Eye Center, Singapore, Singapore; Beijing Visual Science and Translational Eye Research Institute (BERI), Beijing Tsinghua Changgung Hospital Eye Center, Tsinghua Medicine, Tsinghua University, Beijing, China; School of Clinical Medicine, Beijing Tsinghua Changgung Hospital, Tsinghua Medicine, Tsinghua University, Beijing, China; Vanke School of Public Health, Tsinghua University, Beijing, China; Gangarosa Department of Environmental Health, Rollins School of Public Health, Emory University, Atlanta, GA, United States; Department of Medicine, School of Medicine, Emory University, Atlanta, GA, United States; Singapore Eye Research Institute, Singapore National Eye Center, Singapore, Singapore; Duke-NUS Medical School, National University of Singapore, Singapore, Singapore; Byer Eye Institute, Stanford University, Stanford, CA, United States; Singapore Eye Research Institute, Singapore National Eye Center, Singapore, Singapore; Duke-NUS Medical School, National University of Singapore, Singapore, Singapore; Department of Inflammation and Ageing, College of Medicine and Health, University of Birmingham, Birmingham, United Kingdom; Birmingham and Midland Eye Centre, Sandwell and West Birmingham NHS Trust, Birmingham, United Kingdom; Academic Ophthalmology, School of Medicine, University of Nottingham, Nottingham, United Kingdom; Gangarosa Department of Environmental Health, Rollins School of Public Health, Emory University, Atlanta, GA, United States

**Keywords:** oculomics, exposomics, precision environmental health, ocular biomarkers, ocular imaging, multi-omics

## Abstract

Precision environmental health (PEH) is an emerging field that seeks to understand how diverse environmental exposures interact with individual biological and genetic factors to influence health outcomes. While recent advances in exposomics have enabled systematic characterization of the exposome, the integrated compilation of all physical, chemical, biological, and psychosocial influences that affect biology and health, identifying and developing sensitive biomarkers remains a critical challenge. The human eye offers unique potential for non-invasive biomarker discovery. Ocular biomarkers can be utilized not only for diagnostics and therapeutic responses of ocular diseases, but also for monitoring environmental exposures and predicting systemic health outcomes. Retinal imaging modalities such as color fundus photography, optical coherence tomography, and optical coherence tomography angiography capture biomarkers linked to environmental exposures and systemic conditions like cardiovascular and neurodegenerative diseases, a field known as oculomics. Similarly, ocular fluids, such as tears, aqueous humor and vitreous humor, may also reflect pollution-induced oxidative stress and inflammation and systemic health conditions. This paper summarizes current evidence on how ocular biomarkers can bridge environmental exposures and systemic health outcomes, and proposes future research directions using state of the art methodologies such as exposome-wide association studies, high dimensional mediation analysis, and multi-modal foundation models. Despite encouraging progress, significant challenges remain, including the need for large and standardized datasets, rigorous validation, and ethical safeguards to ensure equitable application. Advances in artificial intelligence, including federated learning, alongside global consortium efforts, will be essential to overcome these barriers. Addressing these gaps will unlock the full potential of oculomics and exposomics, advancing the goals of precision environmental health.

## Introduction

The advent of whole genome sequencing has significantly improved our knowledges of role of genes in pathogenesis, yet genes themselves only account less than 10% of the diseases.^1^ The exposome, on the other hand, play a bigger role in biological aging and the development of systemic diseases processes.^2^^,^^3^ Specifically, the exposome refers to the integrated compilation of all physical, chemical, biological, and psychosocial influences—such as air pollution, microplastics, nutrition, psychosocial stress, socioeconomic status, and physical activity—that impact biology.^4-6^ To capture the complexity of the exposome, researchers collect and analyze a wide range of biosamples, including serum, urine, saliva, hair, and other biological matrices, to monitor environmental exposures and their biological effects. While blood samples (serum or plasma) are widely used and particularly valuable for assessing persistent chemicals and circulating metabolites,^7^ they are less suitable for non-persistent compounds with short half-lives, where concentrations in blood may fall below detection limits with high-resolution mass spectrometers-based non-targeted analysis. In such cases, urine or hair can provide more appropriate matrices for capturing exposure profiles.^8^ Advances in mass spectrometry have expanded the ability to detect and annotate both exogenous chemicals and endogenous metabolites across these matrices, though the majority of data are semi-quantitative rather than strictly quantitative.^9^

In recent years, precision environmental health (PEH) has emerged as a new discipline that explores the complex interplay between the exposome and biological systems across the human life course.^10^ Building upon the exposome framework, PEH integrates exposomic data with genomic and molecular information for more personalized health insights. Omics-based biomarkers, including genomics, epigenomics, transcriptomics, proteomics, metabolomics, lipidomics, and microbiomics, provide critical insights into human physiology and pathophysiology, elucidating the molecular mechanisms through which environmental exposures influence human health and contribute to disease development.^11^ Various omics-based biological clocks, such as epigenetic and proteomic clocks, have been proposed, offering tools to quantify biological aging.^12^^,^^13^ While current multi-omics technologies offer powerful tools for deciphering molecular changes, these are often costly, complex and many are invasive, thus limiting the ability to scale the application of these technologies across larger populations in community settings.

Multi-modal ocular biomarkers approach may serve as a novel, cost-effective, and promising complement to the current omics-based biomarkers in PEH. National Institute of Health (NIH) BEST (Biomarkers, EndpointS, and other Tools) resource categorizes biomarkers into different purposes, including diagnostic, response, monitoring, and predictive functions, highlighting their versatility in addressing diverse clinical and research needs.^14^ Though ocular biomarkers have been conventionally used for diagnosing ocular diseases and monitoring treatment response, there is emerging evidence that they can be used to monitor the impact of the exposome on systemic health and predict systemic disease risk over time.^15-19^ Based on synthesis of the emerging evidence, we proposes future directions that integrates ocular biomarker into next generation of PEH research.

## Ocular imaging biomarkers

**Figure 1.**
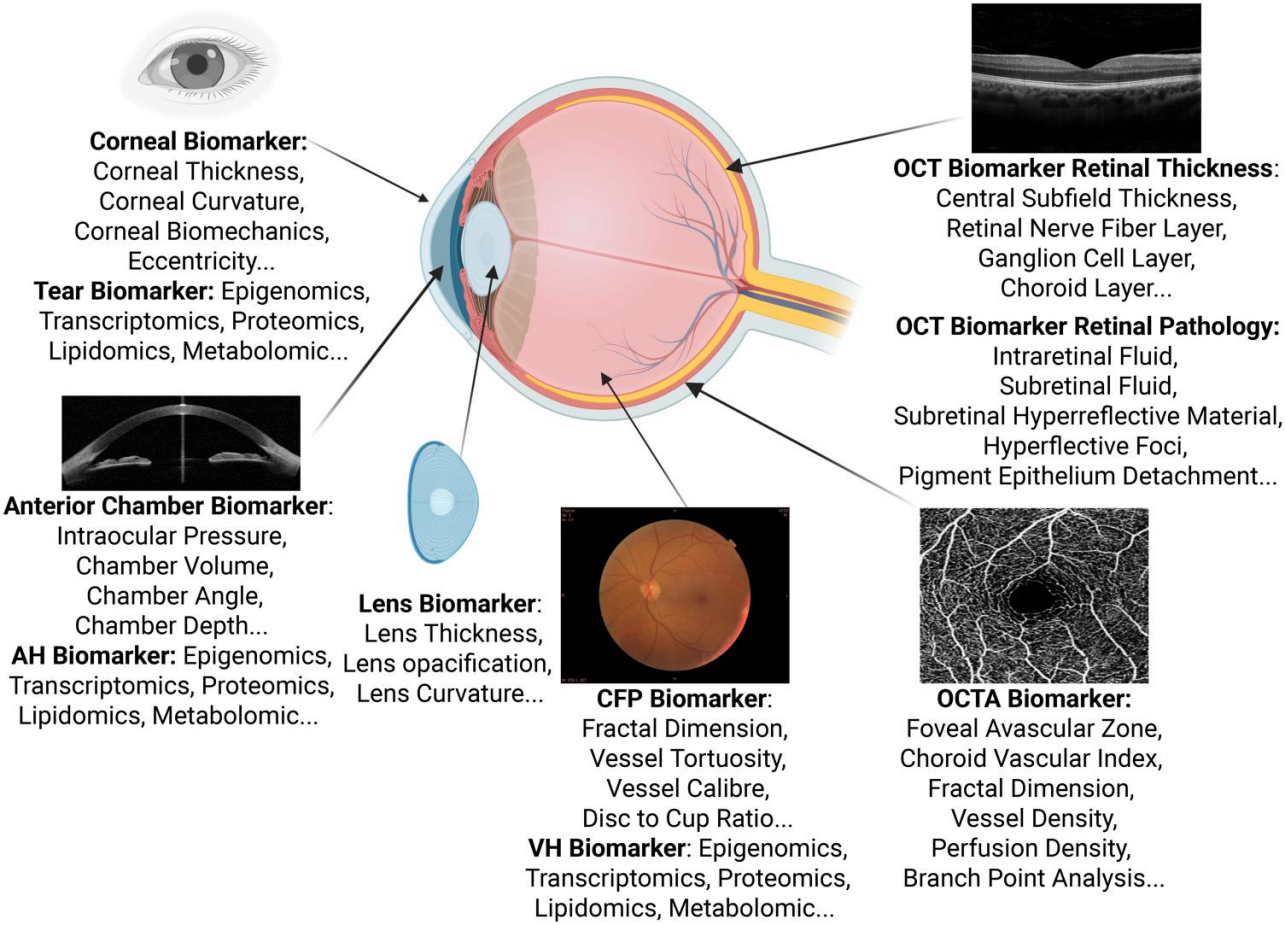
Illustration of various biomarkers based on ocular anatomy. CFP = Color fundus photography; OCT = Optical coherence tomography; OCT-A = Optical coherence tomography angiography; AH = Aqueous humor; VH = Vitreous humor. Created with BioRender.com.

The human eye holds great potential for biomarker discovery due to its unique anatomy and accessibility for non-invasive imaging. (Figure 1) Color fundus photography (CFP) captures high-resolution images of the retina, enabling the assessment of structural biomarkers such as vessel caliber and vessel tortuosity, which reflect vascular health. Optical coherence tomography (OCT) provides cross-sectional and three-dimensional views of the retina, enabling detailed evaluation of specific biomarkers such as retinal layer thickness, the presence of pathological fluid (eg, edema or subretinal fluid).^20^ Optical coherence tomography angiography (OCTA) delivers functional insights into retinal and choroidal blood flow without requiring dye injections, making it particularly valuable for detecting microvascular abnormalities, such as changes in capillary density and foveal avascular zone (FAZ) area.^21^ Advances in artificial intelligence (AI) have enabled automated extraction of quantitative ocular biomarkers with remarkable accuracy and repeatability, making them a scalable and clinically relevant tool for large-scale population health studies.^22-24^

## Imaging biomarker for environmental exposures

Retinal imaging biomarkers are intricately connected to environmental exposures and psychosocial factors, providing valuable insights into how these exposures contribute to microvascular and neurodegenerative changes.^25-27^ Genome-wide association studies (GWAS) suggest that genetic factors account for less than 20% of the variations in retinal vasculature, underscoring the substantial role of environmental and lifestyle factors in shaping retinal microvascular features.^28^  Table 1 summarizes current studies on common environmental exposures and their associated ocular imaging biomarker changes. Notably, similar alterations—such as decreased central retinal artery equivalent (CRAE), increased central retinal vein equivalent (CRVE), and thinning of the retinal nerve fiber layer (RNFL) and ganglion cell inner plexiform layer (GCIPL)—have been observed across diverse exposures, including particulate matter (pm), smoking, and psychosocial stressors. This convergence of findings suggests shared biological pathways through which distinct exposures exert overlapping vascular and neurodegenerative effects.

**Table 1.** Summary of current studies on environmental exposures and ocular biomarkers.

Environmental exposure	Ocular biomarker	Reference
**Imaging biomarker**		
Particulate matter	Decreased CRAE; increased CRVE; RNFL and GCIPL thinning	^25^ ^,^ ^26^ ^,^ ^101-103^
Smoking	Decreased CRAE, DCP vessel density and CVI; increased CRVE; RNFL thinning	^104-108^
Dietary pattern	High carbohydrate food and low fiber intake is associated with decreased CRAE and increased CRVE; heart-healthy diet is associated with decreased tortuosity and increased FD	^32^ ^,^ ^109^ ^,^ ^110^
Psychosocial stress	Increased CRVE; decreased CRAE	^27^
Socioeconomic status	Low SES was associated with low AVR and GCIPL thinning	^111^ ^,^ ^112^
Physical activity	Decreased CRVE; increased CRAE; increased RNFL; smaller FAZ	^33^ ^,^ ^113-115^
**Molecular biomarker**		
Particulate matter	Increased cytokine in tears	^56^ ^,^ ^116^ ^,^ ^117^
Smoking	Decreased tear secretion and increase acute phase reactant proteins; decreased Ascorbic Acid in AH; increased VEGF-A in VH	^116^ ^,^ ^118^ ^,^ ^119^
Psychosocial stress	Increased cortisol in tears	^120^
Physical activity	Decreased oxidative stress marker	^121^

Abbreviations: AH, aqueous humor; AVR, arteriolar-to-venular ratio; CRAE, central retinal artery equivalent; CRVE, central retinal vein equivalent; CVI, choroid vascularity index; DCP, deep retinal capillary plexus; FAZ, foveal avascular zone; FD, fractal dimensions; GCIPL, ganglion cell inner plexiform layer; RNFL, retinal nerve fiber layer; VH, vitreous humor; VEGF, vascular endothelial growth factor.

Mechanistically, chronic exposure to environmental and psychosocial stressors can induce vascular changes through mechanisms like endothelial dysfunction and inflammation. Stress-related hormonal responses impair endothelial nitric oxide (NO) synthesis, a key regulator of vascular tone, and generate reactive oxygen species, which degrade NO and disrupt vascular homeostasis.^29^^,^^30^ Inflammation further mediates these effects, with elevated pro-inflammatory cytokines (eg, IL-6, TNF-α) and C-reactive protein being strongly associated with retinal venular widening, even in children.^30^^,^^31^ These vascular changes may also be indirectly exacerbated by stress-related lifestyle factors such as reduced physical activity and poor dietary habits.^32^^,^^33^ Taken together, these mechanisms provide a biological basis for the observed imaging signatures, reinforcing the value of retinal biomarkers as sensitive indicators of systemic processes—including inflammation, oxidative stress, and microvascular dysfunction.

## Imaging biomarker for systemic health

Retinal biomarkers not only reflect environmental exposures but are also closely linked to systemic diseases.^28^^,^^34^ In 2020, the term oculomics was introduced to describe the study of the associations between ocular biomarkers and systemic health conditions, positioning the retina as an emerging critical component in precision health initiatives.^35^  Table 2 summarizes current studies on ocular imaging biomarkers and systemic health outcomes. Notably, the same biomarkers implicated in environmental exposures—such as increased CRVE, decreased CRAE, and thinning of the RNFL and GCIPL—are also observed across chronic diseases including hypertension, hyperlipidemia, chronic kidney disease (CKD), coronary heart disease, and Alzheimer’s disease and related dementias (ADRD). Phenome-wide association studies have correlated retinal biomarkers with multiple clinical phenotypes and the Eye Biomarker Database consolidates 889 biomarkers across 26 ocular diseases and 939 biomarkers associated with 181 systemic diseases.^36^ Recent advances in AI have enabled the prediction of multiple systemic disease from retinal images with remarkable accuracy.^19^^,^^37-40^

**Table 2.** Summary of current studies on ocular imaging biomarkers and systemic health outcome.

Systemic diseases	Ocular biomarker	Reference
HTN	Increased CRVE; decreased CRAE, tortuosity, SCP and DCP vessel density; choroidal thinning	122-126
HLD	Increased CRVE; decreased CRAE	106,127
T2DM	Decreased SCP vessel density; Increased FAZ; RNFL and GCIPL thinning	128,129
CHD	Increased CRVE; decreased CRAE and FD	130
CKD	Increased CRVE; decreased CRAE and SCP vessel density	131,132
Stroke	Increased CRVE; decreased CRAE and FD	130,133
ADRD	Increased CRVE; decreased CRAE and FD; RNFL and GCIPL thinning; increased FAZ; choroidal thinning; increased CDR	44,134-136

Abbreviations: CRAE, central retinal artery equivalent; CDR, cup to disc ratio; CRVE, central retinal vein equivalent; DCP, deep retinal capillary plexus; FAZ, foveal avascular zone; FD, fractal dimensions; GCIPL, ganglion cell inner plexiform layer; SCP, superficial retinal capillary plexus; RNFL, retinal nerve fiber layer.

The retina’s diagnostic potential lies in its unique ability to integrate and reflect systemic processes due to its shared embryological origin, vascular architecture, and neurovascular connections with other organs. As part of the microcirculation, the retinal vasculature mirrors systemic endothelial health, responding to circulating inflammatory mediators, oxidative stress, atherosclerosis, and hemodynamic changes.^41^^,^^42^ These processes drive vascular remodeling, including arteriolar narrowing, venular widening, and altered vessel tortuosity, while also contributing to neurodegenerative changes, such as amyloid-beta accumulation and tauopathy seen in ADRD.^43^^,^^44^ These pathways are central to the “common soil hypothesis,” implicating shared mechanisms—such as inflammation, oxidative stress, and vascular dysfunction—in multiple chronic diseases, including cardiometabolic diseases, neurodegenerative disorders, and CKD.^45^ These interconnected processes influence retinal and choroidal thickness, vascular permeability, and optic nerve structure, further underscoring the retina’s value as a window into systemic health.

Beyond structural parameters, composite aging biomarkers derived from retinal images provide powerful, non-invasive indicators of systemic aging. The retinal age gap—defined as the difference between predicted retinal age from fundus photographs and chronological age—has been consistently associated with all-cause mortality, cause-specific mortality, and multiple systemic morbidities.^46-48^ Building on this framework, fundus images have also been used to estimate PhenoAge, a validated composite of nine blood-based biomarkers of biological age, giving rise to the retina-derived RetiPhenoAge.^49^^,^^50^ This biomarker has been shown to outperform conventional markers such as telomere length and grip strength in predicting morbidity and mortality, and has demonstrated strong associations with cognitive decline, dementia, cerebral small vessel disease, and proteomic signatures of aging.^51^ Together, these retinal imaging–based biological age markers complement structural parameters by providing scalable, non-invasive, and mechanistically informative indicators of systemic aging across diverse diseases.

## Ocular molecular biomarkers

Beyond imaging biomarkers, the eye’s liquid-filled chambers—tears, aqueous humor (AH), and vitreous humor (VH)—offer a rich reservoir of biomarkers for exposomic analysis. (Figure 1) These fluids are in constant exchange with both the external environment and systemic circulation, thereby capturing signals of external exposures and internal physiological responses. Compared with traditional matrices such as blood, tears can be collected in a minimally invasive manner, making them well suited for temporal monitoring of the exposome.^52^ AH and VH offer complementary information to blood, providing localized insights into ocular pathophysiology and capturing metabolic or inflammatory changes that may not be detectable systemically.

## Molecular biomarker for environmental exposures

Tear, located at the air–tear interface, is particularly sensitive to airborne pollutants.^52^ Studies have shown that tears capture markers of PMs, volatile organic compounds, trace metals, and nicotine within minutes to hours of exposure, highlighting their value for short-term exposure assessment.^52^ More broadly, microplastics (MPs) have now been identified across multiple ocular compartments, including tears, AH, and VH, with their presence associated with alterations in key ocular biomarkers such as tear break-up time, Schirmer’s test scores, intraocular pressure, and vitreous opacities.^53-55^ While analytical methods for micro- and nanoplastic detection are still being refined, converging evidence from these studies suggests that MPs are not confined to the ocular surface but can accumulate throughout different eye matrices, reinforcing the eye’s potential as a unique system for monitoring environmental exposures.

In addition to capturing external exposures, ocular fluids also reflect the body’s internal physiological responses. Tears and AH contain inflammatory and angiogenic mediators that change dynamically with pollutant exposure. Studies have shown significant increases in inflammatory markers like IL-6, IL-8, and vascular endothelial growth factor (VEGF) in tear with increased exposure to air pollution, highlighting its ability to reflect localized and systemic inflammatory effects.^56^ Experimental and animal studies shows that ocular surface exposure to microplastics triggers oxidative stress, inflammation, reduced goblet cell density, and microbial dysbiosis.^57^ Beyond these molecular changes, the ocular surface microbiome has emerged as a key internal exposome. Ambient air pollutants disrupt microbial homeostasis, reducing commensal diversity while enriching pathogenic taxa such as Staphylococcus, Bacteroidia, and Klebsiella, which correlate with goblet cell depletion and chronic ocular inflammation.^58^

## Molecular biomarker for systemic health

Systematic evidence now links tear protein and metabolite changes to a wide spectrum of systemic diseases, including neurodegenerative, autoimmune, and metabolic conditions.^59^ For example, in ADRD, alterations in tear protein composition have been reported, including elevated lactoferrin, which is thought to reflect amyloid-related immune dysregulation and oxidative stress pathways.^59^ In multiple sclerosis, tear proteomics has revealed increased immunoglobulins and pro-inflammatory cytokines, consistent with systemic immune activation and blood–brain barrier dysfunction mirrored at the ocular surface.^59^ Similarly, in type 2 diabetes (T2DM), specific metabolites such as carnitine, tyrosine, and uric acid are elevated in tears, and anti-diabetic drugs like metformin can also be detected, illustrating how tear fluid integrates both disease-related metabolic perturbations and treatment exposure.^60^ Building on this, tear-derived extracellular vesicles (EVs) represent a promising next-generation biomarker platform. EVs protect their protein and RNA cargo from degradation and can cross from the bloodstream into tears, offering a stable and information-rich signal.^61^ They have shown implicated in multiple autoimmune diseases such as Dry Eye Disease, Sjögren’s, graft-versus-host disease.^61^

In addition to tear-based biomarkers, AH and VH provide a complementary perspective, enabling deeper molecular understanding of ocular and systemic aging processes through advanced omic technologies. For instance, using a combination of liquid-biopsy proteomics and AI, researchers have developed proteomic clocks based on the AH and VH specimens that can assess cellular aging within non-regenerative tissues, such as the eye, *in vivo*.^62^ These tools reveal that diseases like ADRD not only alter molecular profiles but also accelerate cellular aging processes in the retina, highlighting the interplay between systemic diseases and ocular aging.^62^

## Research prospects for future

### Apply a systemic approach to study impact of exposome on ocular biomarkers

Despite advances in understanding the role of environmental exposures, the study of their impact on retinal biomarkers remains fragmented. A systematic approach is needed to capture the effects of exposome on retinal structure and function. One promising framework is the Exposome-Wide Association Study (ExWAS).^63^ Inspired by GWAS, which identify associations between genetic variants and disease outcomes, ExWAS adopts a similar agnostic, high-dimensional design to test hundreds of environmental exposures simultaneously for association with a phenotype. Methodologically, ExWAS typically uses logistic or Cox regression with multiple-testing correction.^64^ More recent implementations incorporate machine learning techniques such as shrinkage models, deletion/substitution/addition algorithms for multi-exposure modeling.^65^ Building on ExWAS, Exposome-Wide Interaction Study (ExWIS) extends the framework to model effect modification and biological interactions.^66^ ExWIS systematically tests for interactions between pairs of exposures, or between exposures and genetic risk scores, thereby capturing non-additive and synergistic effects.

Complementary to single-exposure frameworks such as ExWAS, mixture analysis methods are increasingly being used to evaluate the combined effects of multiple, correlated exposures. Methods such as Bayesian Kernel Machine Regression enable flexible modeling of non-linear and interactive effects within mixtures, while Quantile G-Computation provides an interpretable summary estimate of the joint impact of a set of exposures.^67^^,^^68^ By moving beyond single-exposure associations, mixture methods provide a more realistic representation of the exposome and open new opportunities for understanding how complex exposure environments, in interaction with social determinants of health, shape disease risk. Applied to ocular biomarkers, these approaches could clarify how social exposome—including education, food insecurity, financial status, healthcare access—contribute jointly to retinal thinning and vascular caliber changes.^69^

### Explore ocular biomarkers as mediators between environmental exposures and systemic health outcome

Mediation analysis is a statistical and epidemiological approach used to understand the mechanisms through which an exposure influences an outcome. Specifically, it partitions the total effect of an exposure into a direct effect (the portion not explained by intermediates) and an indirect effect (the portion that operates through one or more mediators).^70^ Mediators are typically defined as intermediate variables or biomarkers that lie on the causal pathway between an exposure and an outcome, helping to explain how or why the exposure leads to disease.^70^ By identifying and quantifying these indirect pathways, mediation analysis provides mechanistic insight that complements traditional association studies and can highlight potential intervention targets.^70^ One example of this approach in environmental health is the “meet-in-the-middle” framework, where intermediate biomarkers are evaluated based on their dual association with exposures and outcomes, their plausibility in linking the two, and their reproducibility across studies.^71^

**Figure 2.**
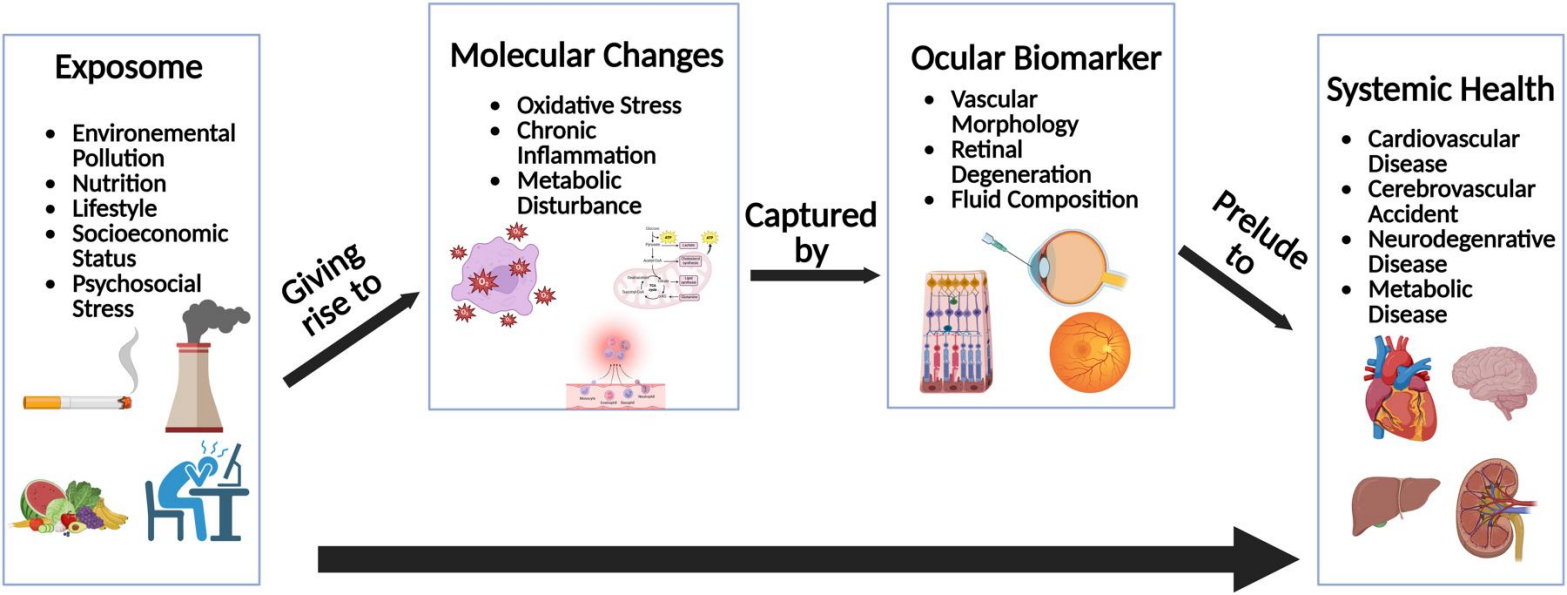
Schematic figure showing the association between exposomics, molecular changes, ocular biomarkers, and systemic health outcome in a conceptual mediation framework. Created with BioRender.com.

Historically, clinical biomarkers and socioeconomic factors have been used as mediators between environmental exposures and systemic health outcomes, with recent efforts expanding to include multi-omics biomarkers.^72^^,^^73^ The development of novel statistical frameworks has enabled high-dimensional mediation analysis, allowing researchers to simultaneously test multiple potential mediators—an approach particularly well-suited for incorporating omics data.^74-77^ While multi-omics approaches are valuable for uncovering complex biological pathways, they are often limited by high costs, technical complexity, the need for advanced equipment, and small sample sizes, which reduce statistical power and generalizability. In contrast, retinal biomarkers present a more accessible and practical alternative. As mentioned in earlier paragraphs, changes in retinal biomarkers such as CRAE, CRVE, and RNFL thickness have each been associated with multiple environmental exposures as well as systemic outcomes. The quantitative shifts in these parameters can therefore act as mediators, providing measurable biological pathways through which the exposome impacts human health. For instance, CRAE maybe a mediator between psychosocial stress and cardiovascular disease outcome, while retinal age gap could be a mediator between dietary inflammation index and all cause mortality.^46^^,^^78^^,^^79^ (Figure 2) Imaging modalities such as OCT and CFP are cost-effective, minimally invasive, highly reproducible, and widely available in clinical settings, enabling larger sample sizes and more robust mediation analyses.^24^

### Integrate multi-modal biomarker for comprehensive mechanistic insight

The crosstalk between structural biomarkers and molecular pathways could reveal the biological underpinnings of observed microstructural changes, offering a window into systemic health dynamics. The IMAGINE study revealed significant correlations between intraretinal fluid features detected through advanced OCT and intraocular cytokine levels, such as VEGF and monocyte chemotactic protein-1 from AH.^80^^,^^81^ To be specific, VEGF levels were associated with increased central subfield thickness and intraretinal fluid volume, highlighting VEGF's role in fluid dynamics and vascular permeability.^80^^,^^81^ This connection to molecular features can potentially help us better understand the molecular mechanisms behind micro-structural changes, thus using eye as model organ to better understanding environmental impact on systemic health. Although the study’s relatively small sample size limits its generalizability, it provides a critical foundation for future research. Larger-scale studies are necessary to validate these findings and expand their applicability.

The rapid advance of foundation models (FMs) presents an exciting opportunity to scale the integration of structural and molecular biomarkers with environmental determinants of health. Unlike conventional models, FMs are trained in a self-supervised manner on massive, heterogeneous datasets and can operate in a multimodal fashion, jointly processing images, omics, clinical text, and geospatial data within a shared representational space.^82-84^ This flexibility makes them uniquely suited for exposome research, where missing data and diverse modalities are common. A recent study demonstrated this potential by predicting both overall and organ-specific biological age from routine health data, with strong associations to mortality that surpassed classical biomarkers.^85^ Current retinal imaging clocks have shown that age gaps derived from fundus and OCT images predict mortality and age-related diseases, while proteomic clocks capture cell-type–specific aging processes not visible on imaging.^46^^,^^62^ Integrating these approaches through a multimodal FM could yield a more comprehensive “Multi-Modal Ocular Aging Clock” that links structural changes with molecular signatures and environmental exposures. Proof-of-concept studies such as OmiCLIP, which align omics and imaging via contrastive learning, illustrate how such integration can be achieved, offering a scalable framework for using the eye as a model organ to uncover how the environment shapes biological aging.^86^

## Limitations & opportunities

One of the most pressing challenges in the field of oculomics is the need for standardized “big” data. The power of oculomics studies largely stems from their reliance on vast datasets, often incorporating tens of thousands of retinal images. Similarly, ExWAS usually requires a large sample size to yield statistically significant results. However, the intersection of these two fields presents a significant hurdle: very few datasets currently exist that include both comprehensive exposomics data and detailed retinal imaging on a scale sufficient to conduct robust analyses. Table 3 summarizes current datasets current datasets with exposome and ocular imaging data. Notably, two major large scale cohorts for epidemiological research in the United States, All of Us, and Million Veteran Program currently both do not collect or provide retinal imaging data for research purpose.^87^^,^^88^ UK Biobank is so far the only national biobank that incorporates multi-modal ocular imaging, in an organized and easily retrievable format.^89^ Its rich modalities of data, comprehensive coverage of diseases, and large sample size have made it an essential part of most of the exposomics and oculomics studies nowadays. A few disease-specific cohorts, such as the Atherosclerosis Risk in Communities (ARIC) study and the Chronic Renal Insufficiency Cohort (CRIC), include ocular imaging data.^90^^,^^91^ However, large exposome cohorts such Human Early Life Exposome (HELIX) and Personalized Environment and Genes Study (PEGS) do not collect retinal imaging as one of their data modalities.^64^^,^^92^ The scarcity of such large, integrated datasets limits the ability to fully explore the potential connections between environmental exposures and retinal biomarkers, which in turn hinders our capacity to uncover novel insights into disease mechanisms. To overcome this limitation, multi-center collaborations and international consortium initiatives will be essential. The National Heart, Lung, and Blood Institute for example convened a workshop in 2022 that emphasized the importance of standardized retinal imaging protocols, interoperable data structures, and scalable analytics to advance the use of retinal biomarkers in cardiovascular and systemic disease research.^42^

**Table 3.** Summary of current cohorts with environmental exposures and ocular imaging data.

Cohort	Baseline population	Exposure data		Ocular imaging	
		Environmental sensor	Biospecimen	CFP	OCT
**Large scale national cohort**					
UKBiobank^89^	500 000 individual aged 40-69 years in UK	+	+	+	+
All of US^88^	1 million individuals in US	+	+	+	–
Million Veteran Program^87^	1 million military veterans in US	–	+	–	–
Rotterdam Study^137^	15 000 individuals in Netherlands	+	+	+	+
China Kadoorie Biobank (CKB)^138^	512 000 adults aged 30-79 years from China	+	+	–	–
Human Phenotype Project^139^	100 000 individuals globally	–	+	+	–
PRECISE SG100K^140^	100 000 individuals aged 21-84 in Singapore	–	+	+	+
**Medium to large cohort on specific diseases**					
Artificial Intelligence Ready and Exploratory Atlas for Diabetes Insights (AI-READI)^141^	4000 individuals with and without diabetes from US	+	+	+	+
Atherosclerosis Risk in Communities (ARIC)^142^	15 800 individuals (aged 45–64 years) in US	+	+	+	–
Singapore Epidemiology of Eye Disease (SEED)^143^	over 10 000 Malay, Indian and Chinese individuals aged over 40 years in Singapore	–	+	+	+
Beaver Dam Eye Study^144^	5000 individuals aged 43-84 years from Beaver Dam, Wisconsin	–	+	+	–
Blue Mountain Eye Study^145^	3600 individuals aged > 50 years from Australia	–	+	+	–
Multi-Ethnic Study of Atherosclerosis^146^	6800 individuals aged 45-84 years from four ethnic groups (White, African American, Hispanic, and Chinese) in US	+	+	+	–
Beijing Eye Study^147^	4439 residents aged ≥ 40 years in Beijing, China	–	+	+	+
Chronic Renal Insufficiency Cohort (CRIC)^148^	5500 individuals in US	–	+	+	–

Abbreviations: CFP, Color fundus photography; OCT, Optical coherence tomography.

Another critical challenge is the lack of standardized exposomics profiling. Currently, even if researchers identify several cohorts with rich environmental exposome data, inconsistencies in biometrics, sample collection and storage protocols, and lab analytical platforms can make it difficult to integrate the omics data across cohorts. For instance, the composition of tears has high temporal variability, making it difficult to compare data across different studies.^93^^,^^94^ To overcome these barriers, the establishment of internationally accepted guidelines for data normalization, harmonization, and sharing is urgently needed. The Banbury Exposomics Consortium has made infrastructure development and the establishment of data standards for harmonization one of top priorities for exposome research.^95^ Beyond biospecimen analysis, questionnaire-based data collection also requires rigorous standardization. Although most cohorts incorporate questionnaires, these are rarely uniform and are often tailored for specific study objectives rather than capturing the full breadth of the exposome. A notable example of efforts to address this challenge is the PEGS, a large and demographically diverse North Carolina-based cohort.^64^^,^^96^ PEGS collects exposome data through multiple surveys, including the Health and Exposure Survey, the Internal Exposome Survey, and the External Exposome Survey, which together capture over 1000 variables on lifestyle, occupational and residential exposures, medication use, diet, sleep, stress, and infectious diseases.^64^^,^^96^

The collection and management of such extensive datasets also raise significant concerns about data privacy, particularly when data are gathered from multiple sites. These exposomics and retinal imaging data are highly sensitive personal information, making it crucial to implement robust data protection measures to ensure confidentiality. The use of AI algorithms to analyze retinal images may introduce biases if the training data is not representative of diverse populations.^97^ Beyond population diversity, it is equally important to ensure that the data encompasses a wide range of systemic and ocular conditions, as these significantly influence retinal changes. A lack of representation in such conditions could hinder a comprehensive understanding of their effects and interactions. This gap may further exacerbate healthcare disparities, particularly for individuals with limited access to advanced ocular imaging technologies, who may be excluded from the analysis.^97^ Addressing these ethical and logistical challenges is crucial to ensure that the integration of exposomics and oculomics is both scientifically robust and equitable.

One potential solution to address data privacy concerns is through approaches such as federated learning, a machine learning technique that enables multiple institutions or devices to collaboratively train a shared model without transferring raw data to a central repository.^98^^,^^99^ Instead, the data remains local, and only model updates, such as gradients or weights, are shared and aggregated. This approach can enhance privacy while still enabling comprehensive analysis. In parallel with these privacy-preserving strategies, global collaborative efforts are equally important to tackle the pressing issue of diversity and representation in ocular imaging datasets.

The GlobalRetFound Consortium is one initiative designed to address the lack of diversity in ocular imaging data.^100^ It aggregates contributions from over 100 countries and combines synthetic data generation with selective real data to build a more representative global dataset. By minimizing the need for direct raw data transfer, the consortium supports data privacy while expanding demographic and geographic diversity. Similar international consortia will be essential to integrate oculomics and exposomics, ensuring that future precision environmental health research is both inclusive and equitable.

## Conclusion

Ocular biomarkers hold great potential to advance PEH research because they are non-invasive, scalable, and capable of capturing both structural and molecular signatures of systemic processes. Retinal imaging modalities such as CFP, OCT, and OCTA provide reproducible measures of vascular health, neurodegeneration, and biological aging, while ocular fluids capture molecular signatures of environmental exposures and systemic responses. Together, they offer a cost-effective and accessible complement to traditional omics-based biomarkers. Looking forward, novel research methodologies—including ExWAS, high dimensional mediation analysis, and multimodal foundation models—offer exciting opportunities to better integrate ocular biomarkers with environmental data. Nonetheless, significant challenges remain. The scarcity of large datasets linking exposome and ocular data, lack of standardized profiling methods, and issues of data privacy and equity limit progress. Addressing these gaps through international collaboration, harmonization of data collection, and equitable technology deployment will be crucial. By overcoming these barriers, ocular biomarkers can become a cornerstone of PEH, enabling earlier detection, targeted prevention, and healthier longevity.

## Data Availability

No new data were generated or analyzed in support of this research.
